# Opportunistic gill infection is associated with TiO_2_ nanoparticle-induced mortality in zebrafish

**DOI:** 10.1371/journal.pone.0247859

**Published:** 2021-07-20

**Authors:** Chiao-Yi Huang, Wei-Sheng Yu, Geng-Chia Liu, Shih-Che Hung, Jen-Hsiang Chang, Jen-Che Chang, Chia-Liang Cheng, Der-Shan Sun, Ming-Der Lin, Wen-Ying Lin, Yin-Jeh Tzeng, Hsin-Hou Chang

**Affiliations:** 1 Department of Molecular Biology and Human Genetics, Tzu-Chi University, Hualien, Taiwan; 2 Tzu-Chi Senior High School Affiliated with Tzu-Chi University, Tzu-Chi University, Hualien, Taiwan; 3 Institute of Medical Sciences, Tzu-Chi University, Hualien, Taiwan; 4 Department and Graduate School of Computer Science, National Pingtung University, Pingtung, Taiwan; 5 Stella Maris High School, Hualien, Taiwan; 6 Department of Physics, National Dong Hwa University, Hualien, Taiwan; University of Southern Denmark, DENMARK

## Abstract

The large amounts of engineered titanium dioxide nanoparticles (TiO_2_NPs) that have been manufactured have inevitably been released into the ecosystem. Reports have suggested that TiO_2_ is a relatively inert material that has low toxicity to animals. However, as various types of NPs increasingly accumulate in the ocean, their effects on aquatic life-forms remain unclear. In this study, a zebrafish model was used to investigate TiO_2_NP-induced injury and mortality. We found that the treatment dosages of TiO_2_NP are positively associated with increased motility of zebrafish and the bacterial counts in the water. Notably, gill but not dorsal fin and caudal fin of the zebrafish displayed considerably increased bacterial load. Metagenomic analysis further revealed that gut microflora, such as phyla *Proteobacteria*, *Bacteroidetes*, and *Actinobacteria*, involving more than 95% of total bacteria counts in the NP-injured zebrafish gill samples. These results collectively suggest that opportunistic bacterial infections are associated with TiO_2_NP-induced mortality in zebrafish. Infections secondary to TiO_2_NP-induced injury could be a neglected factor determining the detrimental effects of TiO_2_NPs on wild fish.

## Introduction

Titanium dioxide (TiO_2_) forms naturally as the well-known minerals rutile, anatase, and brookite phases. Industrial production of TiO_2_ occurs at a large scale, and an estimated 165,050,000 metric tons of TiO_2_ were produced worldwide between 1916 and 2011 [1]. Products containing TiO_2_ nanoparticles (TiO_2_NPs), such as sunscreen, cosmetics, paints, and semiconductors, are widely manufactured in various industries [2, 3]. For example, upon ultraviolet (UV) irradiation, the photocatalytic properties of TiO_2_ in the form of anatase enable it to catalyze H_2_O to release reactive oxygen species [4–6], which can be used in disinfectants and self-cleaning products [4, 5, 7–12]. The UV-shielding property of TiO_2_ has led to its use in skin-protecting sunscreens and cosmetics [13–15]. Although TiO_2_ is a vital component of these everyday products, its use means that human-made TiO_2_NPs will inevitably be released into the ecosystem. Experimental data–based safety guidelines for the release of TiO_2_NPs into fresh or salt water are not yet available. For example, neither the United States Environmental Protection Agency Aquatic Life Criteria nor the United Kingdom Environmental Quality Standards clearly indicate specific standards for the release of TiO_2_NPs [2]. Contamination by TiO_2_NPs has been proven to negatively affect aquatic life-forms, primarily through direct NP-induced toxicity [16, 17], although the other indirect damages remain unclear.

Zebrafish (*Danio rerio*) are widely used for vertebrate models in the study of diseases and is increasingly being used in preclinical and toxicological studies [16]. As many fundamental cellular pathways involved in the response to toxicants or stresses are highly conserved between the zebrafish and mammals, it has been considered as a ‘gold standard’ for environmental toxicity assessment [18]. More recently, it has been demonstrated to be a useful model for evaluating the environmental health and safety impacts of engineered nanomaterials and nanoscale products, which are increasingly being produced as a result of developments in nanotechnology [17–26]. In addition, benefited by the size and transparent body, zebrafish could be used to observe the impact of NPs on the induction of reactive oxygen species and apoptosis pathways at the cellular level [17, 20, 23, 24, 26, 27]. Furthermore, various analysis methods and high-throughput screening systems have been developed for use in toxicological evaluations [28, 29].

The direct effects of NP-induced toxicity have been revealed by recent studies [19–27, 30–34]. However, the NP-induced collateral damages, such as interactions between injured fish and surrounding microorganisms, have not been considered, and their role remains unclear. We hypothesized that NP-induced injuries are desired conditions for the amplification of those opportunistic infectious bacteria, which may be involved in NP-induced detrimental effects in aquatic life forms. Accordingly, in this study, we investigated the progression of photocatalysis-independent TiO_2_NP-induced injury using a zebrafish model. We found that TiO_2_NP-induced opportunistic bacterial gill infections play a critical role in TiO_2_NP-induced zebrafish death. Potential implications are also discussed.

## Materials and methods

### Chemicals and TiO_2_NPs

The chemicals used in this study were purchased from Sigma-Aldrich (St. Louis, MO, USA). To prepare the stock solutions of 1 mg/mL Degussa P25 (Evonik Degussa, Essen, Germany) TiO_2_NPs (21 ± 5 nm) [4, 8, 35], the NPs were dispersed in distilled deionized water under sonication (50 W/L, 40 kHz) for 20 min. Test TiO_2_NP solutions were prepared immediately before use through dilution of the stock solutions with distilled deionized water and sonication (50 W/L, 40 kHz) for 20 min. In our studies, the particle size distributions and ζ-potential were estimated using the dynamic light scattering method (DLS) with a Zetasizer Nano ZS (Malvern Instruments, Malvern, UK) [36, 37]. The averaged ζ-potential of the used TiO_2_NPs in solution was 22.08 ± 0.32 mV, with pH value around 5.35–5.45. The TiO_2_NPs obtained (from Sigma Aldrich) has an averaged particle size about 20 nm according to company specification, but in medium the particles aggregated and measured to be 100 nm.

### Zebrafish maintenance and experimental procedure

Adult wild zebrafish were used in the experiment and were kept in a semistatic system with charcoal-filtered tap water (pH 7.0–7.4) at 28 ± 0.5°C as recommended in a previous study [38], with a 12-h light–12-h dark (12L:12D) photocycle (Classictone incandescent lamp, Philips; Taiwan, without illuminating UV to avoid UV-induced photocatalysis [5, 11, 12, 33, 39, 40]). The fish were fed newly hatched brine shrimp and pellet food (Zeigler Brothers, Gardners, PA, USA) and were kept in 30-L glass tanks with 20 L of water per tank. The zebrafish undergoing testing were exposed to TiO_2_NPs in well water for 21 days, and the mortality was recorded each day. The Kaplan Meier curves are plotted using the Online Application for the Survival Analysis of Lifespan Assay (http://sbi.postech.ac.kr/oasis) [41–44]. Fish from three of the 3-L tanks were selected for behavior analysis, in which the swimming speed of the zebrafish was analyzed using Kinovea software (version 0.8.24; available at http://www.kinovea.org/) in accordance with methods used in previous studies [45, 46]. After the experiment, the fish were euthanized with an overdose of tricaine methanesulfonate MS-222 (0.03%; Sigma-Aldrich). The gill and fin tissue samples were excised, weighed, cut into small pieces, and homogenized with 100 μL of phosphate-buffered saline at 4°C. Next, the 30-μL tissue homogenates were placed on luria broth (LB) agar plates (BD Difco LB Agar; Becton Dickinson, Taipei, Taiwan), using standard bacterial culture protocols [39, 47]. The bacteria colony–forming unit on the plates was determined at a 24-h incubation period at 37°C according to previously described methods [39]. The zebrafish (AB strain) used in the present study were obtained from the zebrafish facility at the Laboratory Animal center of Tzu Chi University. Institutional Animal Care and Use Committee of Tzu Chi University approved all animal experiments in this study (approval ID: 105060).

### Ethics statements

All methods on the collection and analyses of zebrafish samples were performed in accordance with Animal Protection Act, Taiwan, and were approved by the Institutional Animal Care and Use Committee of Tzu-Chi University, Hualien, Taiwan (approval ID: 105060).

### Microbiome analysis

#### DNA preparation

The bacterial genomic DNA was extracted from the 200-mg frozen gill samples with a QIAamp Fast DNA Stool Mini Kit (Qiagen, Venlo, Netherlands). After isolation, the DNA yield was approximately 1–2 μg. The DNA sample was stored at −20°C before polymerase chain reaction (PCR) amplification.

#### PCR amplification

The DNA samples were adjusted to 25 μg/mL. Forward and reverse primers that were complementary upstream and downstream of the V3-V4 region of 16S were designed with Illumina overhang adapters, and used to amplify templates from bacterial genomic DNA. The following 16S amplicon PCR primer sequences were shown: forward, 5’-TCG TCG GCA GCG TCA GAT GTG TAT AAG AGA CAG CCT ACG GGN GGC WGC AG-3’; reverse, 5’-GTC TCG TGG GCT CGG AGA TGT GTA TAA GAG ACA GGA CTA CHV GGG TAT CTA ATC C-3’. The PCR products were then purified with a GenepHlow Gel/PCR purification kit (Geneaid, New Taipei City, Taiwan).

#### Index PCR and clean up

The Illumina sequencing adapters and dual indices were attached to the PCR products using the Nextera XT Index Kit (Illumina Inc, San Diego, CA, USA). Next, AMPure XP beads were used to clean up the final libraries, and the expected size on the Bioanalyzer trace of the final libraries was approximately 630 bp.

#### Normalization and sequencing

Libraries were normalized and pooled and then sequenced on the MiSeq System using v3.0 reagents (paired-end 250 bp, Illumina Inc).

#### Data analysis

Because microbiota profiling using specific hypervariable regions of 16S ribosomal RNA cannot reach taxonomic levels lower than the family or genus level [48], we obtained the results at the family level. The microbiome analysis data is available at NCBI Sequence Read Archive, Accession: SRR14876120.

#### Statistical analysis

The mortality of zebrafish was calculated using Online Application for the Survival Analysis of Lifespan Assay (http://sbi.postech.ac.kr/oasis) [41–44]. A *t* test was used to assess the statistical significance of differences in antimicrobial effects. A *P* -value of less than 0.05 (*P* < 0.05) was considered significant. The statistical tests were performed and output to graphs using Microsoft Excel (Microsoft, Taipei, Taiwan) and SigmaPlot (Systat Software, Point Richmond, CA, USA).

## Results

### Increased TiO_2_NP levels in water reduced motility and induced mortality in adult zebrafish

**Fig 1:**
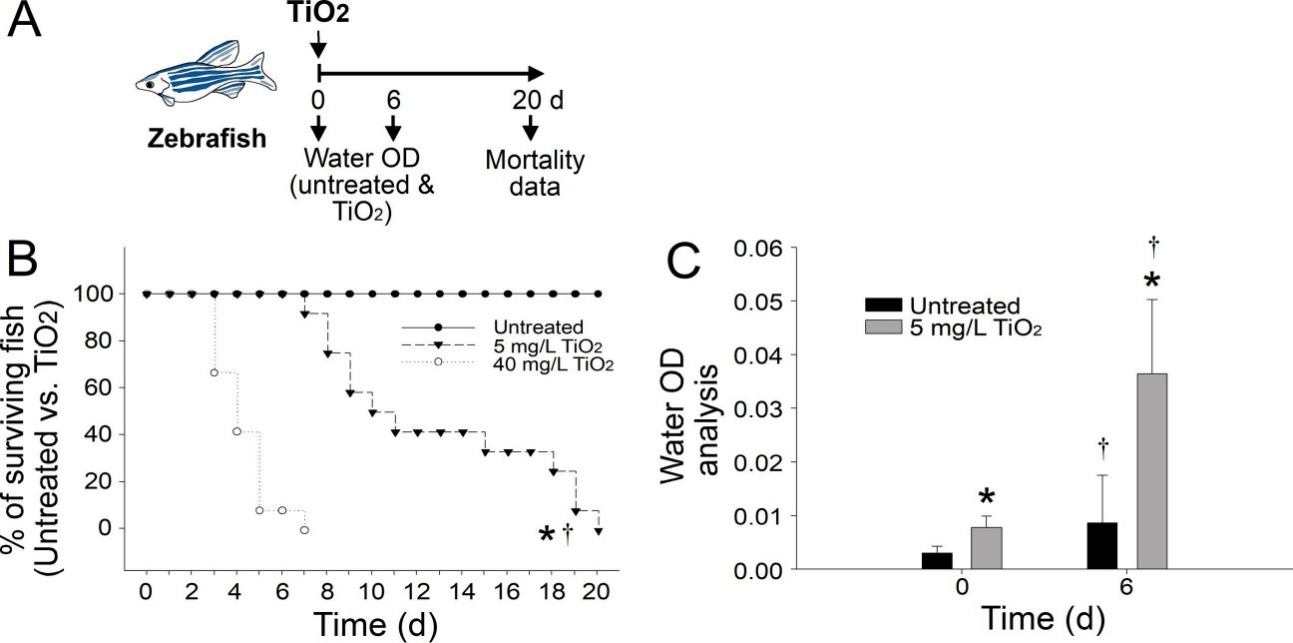
Zebrafish mortality is associated with increased TiO_2_NP levels in the water. (A) Experiment outline. (B) TiO_2_NP dose–dependent induction of mortality in zebrafish. (C) Increase in water OD after the addition of TiO_2_NPs. * *P* < 0.05, compared with the untreated group; ^†^ *P* < 0.05, compared with the 40 mg/L treatment groups (B); * *P* < 0.05, compared with the untreated groups; ^†^ *P* < 0.05, compared with the groups at day 0 (C) n = 16.

Two doses (5 and 40 mg/L) of TiO_2_NPs were used in the investigation of TiO_2_NP-induced mortality in adult zebrafish (Fig 1). We found that the 5 and 40 mg/L doses of TiO_2_NPs induced 100% mortality in adult zebrafish after 20 and 7 days, respectively (Fig 1A experiment outline; Fig 1B).

**Fig 2:**
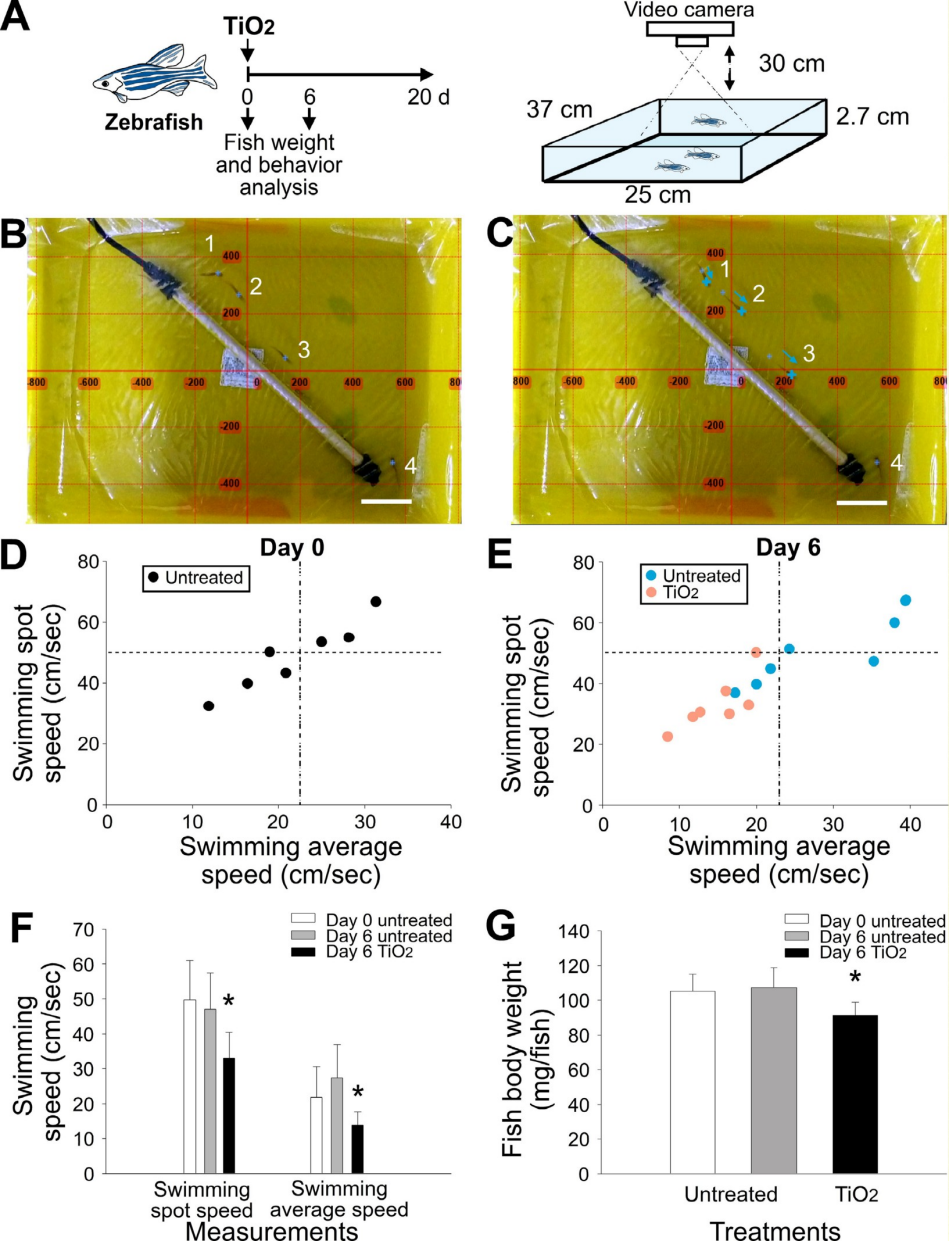
Analysis of zebrafish motility in water containing TiO_2_NPs. (A) Experiment outline and settings. (B, C) Sequential images of zebrafish in tanks with water containing TiO_2_NPs. (D) 2D plots of zebrafish spot swimming speed versus average swimming speed at day 0 (untreated) and (E) day 6 (with or without TiO_2_NPs); dashed lines represent the average spot speed and average swimming speed of the groups at day 0. (F) Statistical analysis of zebrafish swimming speed and (G) body weight under various conditions. * *P* < 0.05, compared with the untreated groups at day 6. n = 7.

Although no mortality had occurred by day 6, the absorbance of water in the zebrafish tanks was considerably increased compared with that of the untreated control groups (Fig 1C, untreated groups vs. groups treated with 5 mg/L TiO_2_NPs). For this reason, we hypothesized that the zebrafish in the groups treated with 5 mg/L TiO_2_NPs may have been subject to TiO_2_NP-induced injury prior to mortality, and the resulting release of blood or tissue fluids would subsequently cause optical-density changes in the water. Consequently, to assess the physical condition of the fish, the motility (Fig 2A–2C, experiment setting; Fig 2D–2F) and body weight (Fig 2G) of the fish in the untreated groups were compared with those of the groups treated with 5 mg/L TiO_2_NPs prior to mortality (day 6 in the 5 mg/L TiO_2_NPs groups). We found that when compared with the untreated groups, the groups treated with 5 mg/L TiO_2_NPs exhibited remarkably reduced motility, as indicated by decreases in both the average swimming speed and spot swimming speed of the zebrafish (Fig 2D–2F). These results indicated that the fish in the groups treated with 5 mg/L TiO_2_NPs were harmed by the TiO_2_NPs.

### TiO_2_NP-induced mortality in zebrafish is associated with increased gill bacterial counts

**Fig 3:**
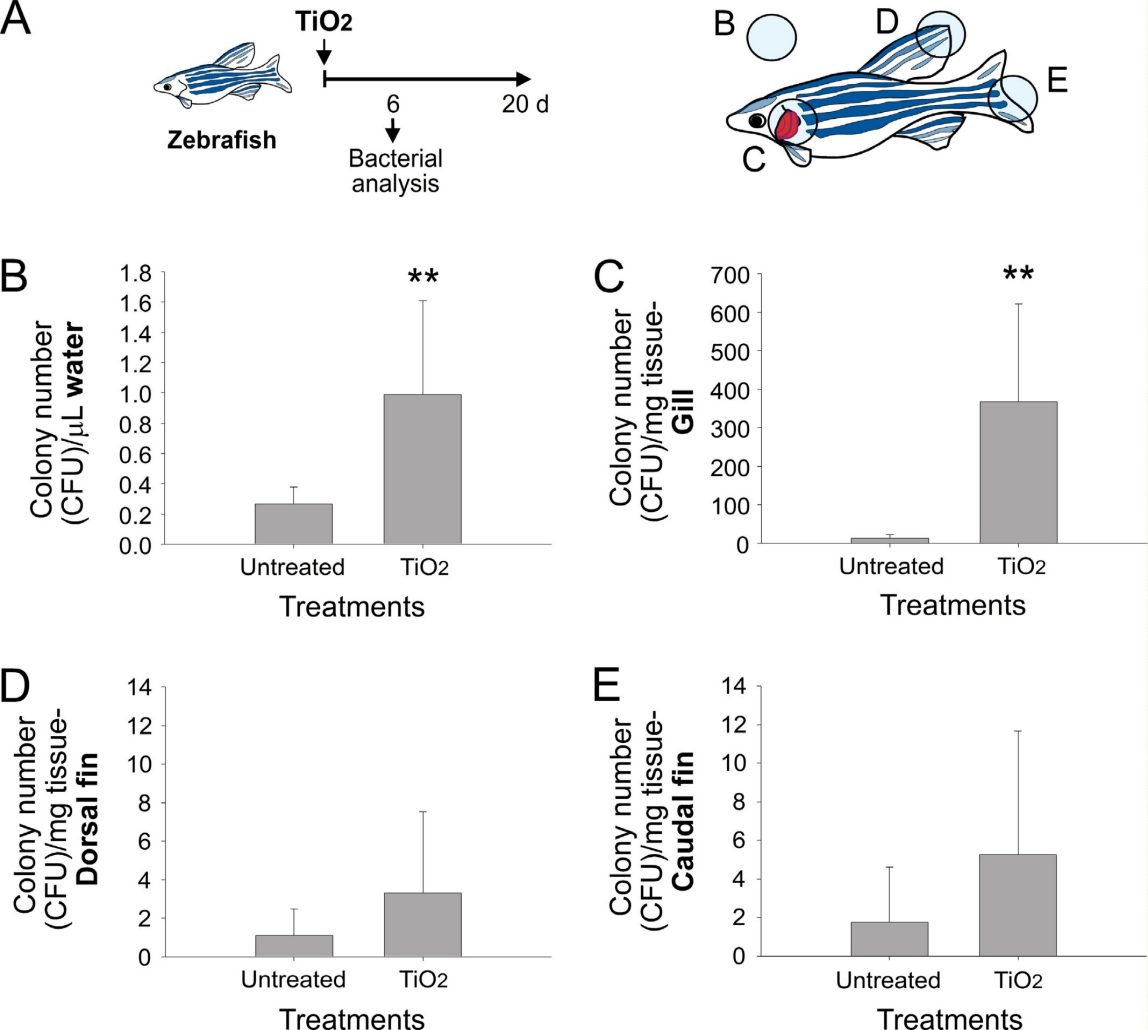
Analysis of the relative number of bacteria found in the water and zebrafish tissue samples. (A) Experiment outline and (B) sampling positions for bacterial culture. The culturable bacterial number in (B) the water, (C) zebrafish gill, (D) dorsal fin, and (E) caudal fin after analysis with the plating method. ** *P* < 0.01, compared with the untreated control groups.

Pure TiO_2_NPs do not display antibacterial property unless with exposure of UV [4, 10–12, 33, 39, 40, 49]. To maintain zebrafish, incandescent lamps were used as a light source, which does not irradiate UV light to induce photocatalysis, and thus the TiO_2_NPs will not exert antibacterial effects in this experimental condition [5, 11]. To further investigate whether the increased water optical density (OD) was caused by an overgrowth of bacteria, we analyzed bacteria colonies from the water and the zebrafish tissues by using a plating method (Fig 3 and S1 Fig in S1 Data). We found that the TiO_2_NP treatments substantially increased the number of bacteria colonies in the water samples. Among the analyzed zebrafish tissues, including those from gills, dorsal fins, and caudal fins, only the gill samples exhibited a significant increase in the number of bacteria colonies (Fig 3; ** *P* < 0.01, TiO_2_-treated groups vs. untreated groups). This result indicates that TiO_2_NP-induced zebrafish injury involves gill infection.

**Fig 4:**
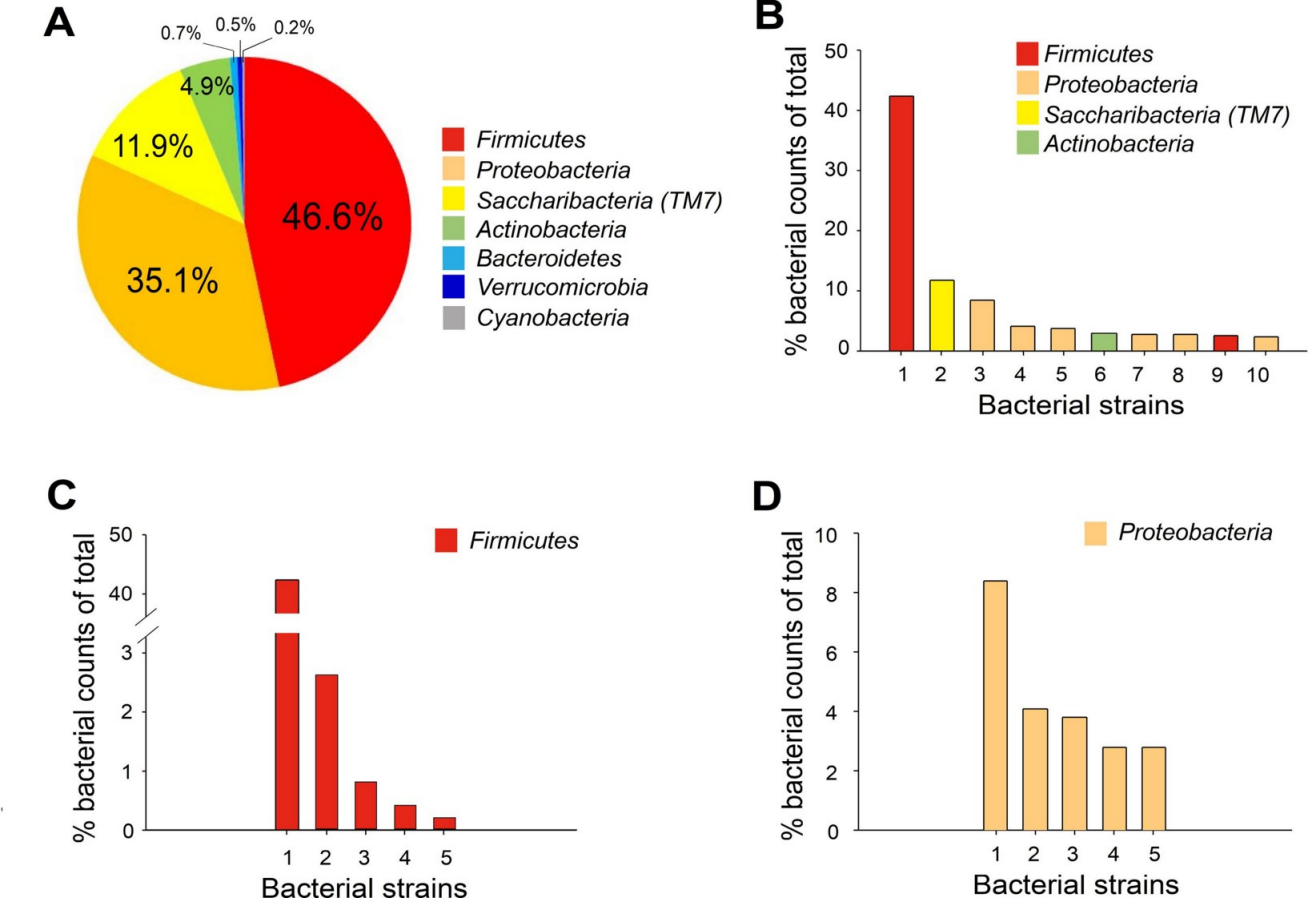
Metagenomic analysis of bacterial communities in the gill samples of zebrafish with TiO_2_NP-induced injury. (A) Relative abundance (% relative to the total) of the bacteria populations calculated for specific hypervariable regions of 16S ribosomal RNA through new-generation sequencing analyses. (B) Relative abundance (% counts of total) of the top 10 overall bacteria families (listed in the following paragraph). (C) Top 5 bacteria families in the *Proteobacteria* phylum (most abundant phylum; listed below). (D) Top 4 bacteria families in *Bacteroidetes* phylum (second abundant phylum; listed below).

Because the plating method can only reveal culturable bacteria [50, 51], metagenomic analysis was performed to investigate the entire spectrum of the bacteria population in the infected zebrafish gills. The relative abundance (% relative to the total) of the bacteria populations was analyzed in specific hypervariable regions of 16S ribosomal RNA through new-generation sequencing analyses (Fig 4 and S2 Fig in S1 Data). We found that the phyla *Proteobacteria*, *Bacteroidetes*, and *Actinobacteria*, all of which are bacteria found in normal zebrafish gut microbiomes [52–55], accounted for more than 95% of the total bacteria counts (Fig 4). Because the fish culture conditions did not include pathogens, these results indicate that TiO_2_NP-induced zebrafish gill injury is associated with opportunistic infection of the gut normal flora.

## Discussion

Zebrafish have been used to study engineered nanomaterials and NPs in various fields, such as biomedical research [38, 56–58] and studies on environmental health and safety [59, 60]. Most relevant studies have reported that TiO_2_NPs are toxic and induce mortality in zebrafish embryos but are less toxic and cause less mortality in adult zebrafishes; this finding is likely attributable to the fact that these studies have tended to focus only on observations of acute stimulation [18, 61–64]. In addition, although researchers have concluded that the injury and mortality in the tested zebrafish were attributable to the chemical and physical properties of NPs [18, 61–64], damage caused by secondary infections has yet to be investigated.

**Fig 5:**
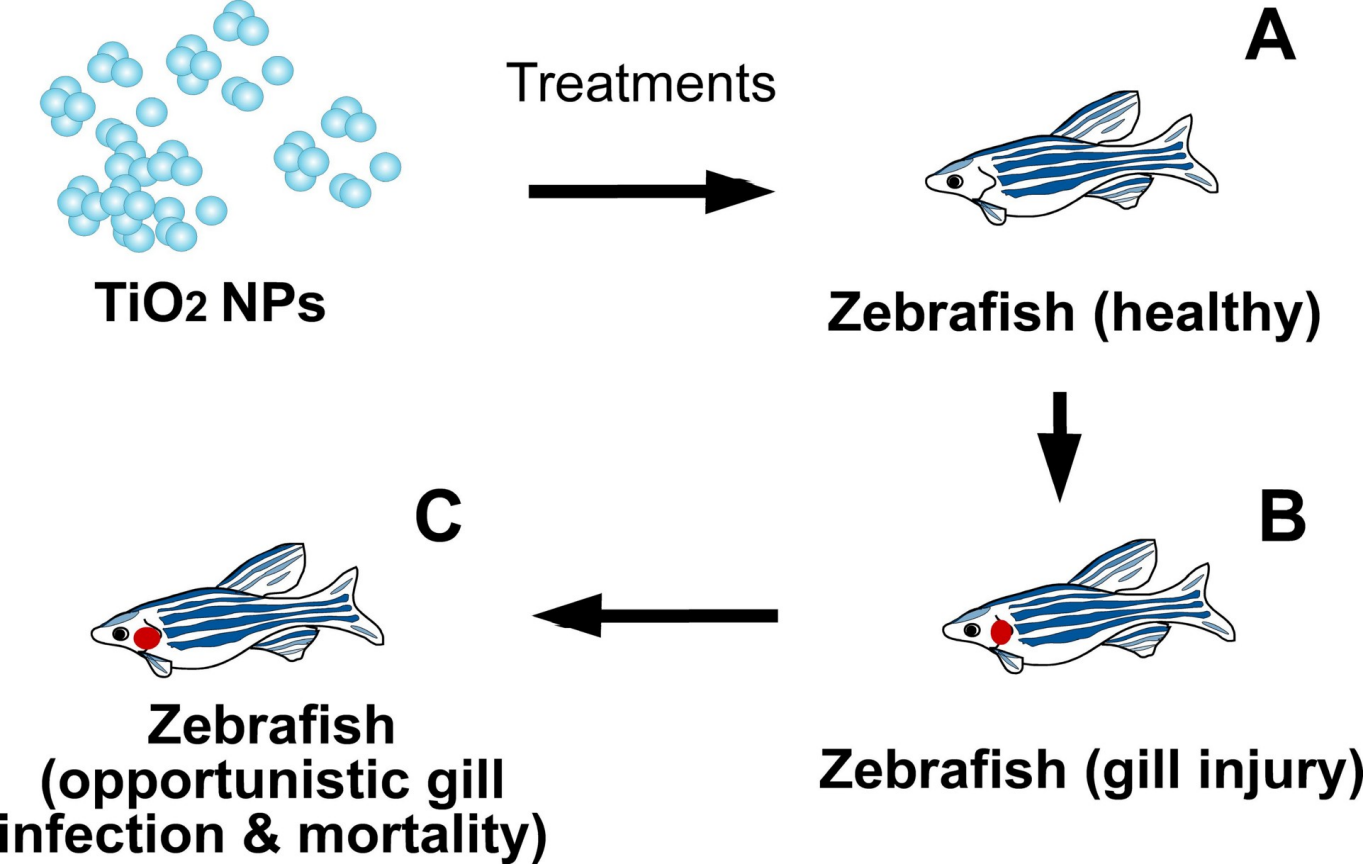
A model for putative role of opportunistic gill infection in TiO_2_NP-induced mortality in zebrafish. After TiO_2_NP treatments, the gill of healthy zebrafishes (A) become injured (B). The gill injury further leads to opportunistic infection and increased mortality in zebrafish (C).

In our study, we found that treating adult zebrafish with 1–3 weeks of relatively low doses (5–40 mg/L) of TiO_2_NPs led to reduced motility, reduced body weight, and increased mortality. Additionally, we also found that the adverse effects of TiO_2_NPs were associated with gill infection. Accordingly, we postulate a hypothetical model, in which TiO_2_NPs-induced gill injury the fish at the first stage, while opportunistic gill infection may further exacerbate the injury and then lead to mortality (Fig 5). This suggests that wild fish inhabiting rivers, lakes, and oceans may not immediately die upon exposure to water contaminated by TiO_2_NPs but that subsequent opportunistic infections determine the survival of aquatic life-forms subject to NP-induced injury.

Through metagenomic analysis, we discovered that bacteria found in the gill samples of zebrafish with NP-induced injury, such as the phyla *Proteobacteria*, *Bacteroidetes*, and *Actinobacteria*, accounted for more than 95% of the total bacteria count; these types of bacteria are all found in normal zebrafish gut microflora [52–55]. This result is likely attributable to the fact that the zebrafish in the experiment were kept under pathogen-free conditions; no pathogens other than normal flora were present. However, considering the fact that a larger number of pathogenic microorganisms coexist in the normal habitats of wild aquatic life-forms, TiO_2_NPs may theoretically be more toxic to wild fish and lead to higher mortality. As an increasing amount of NPs are released into rivers, lakes, and oceans [65, 66], infections in wild fish and other aquatic life-forms as a result of NP-induced injury represent a serious issue worthy of further investigation.

### Top 10 bacteria families and abundance (% total counts) in all detections (Fig 4B) (k: Kingdom; P: Phylum; c: Class; o: Order; f: Family)

1. k__Bacteria;p__Proteobacteria;c__Gammaproteobacteria;o__Betaproteobacteriales;f__Burkholderiaceae;g__Sphaerotilus.
2. k__Bacteria;p__Bacteroidetes;c__Bacteroidia;o__Sphingobacteriales;f__env.OPS_17;g__Ambiguous_taxa;s__Ambiguous_taxa
3. k__Bacteria;p__Proteobacteria;c__Gammaproteobacteria;o__Aeromonadales;f__Aeromonadaceae;g__Aeromonas.
4. k__Bacteria;p__Actinobacteria;c__Actinobacteria;o__Corynebacteriales;f__Mycobacteriaceae;g__Mycobacterium.
5. k__Bacteria;p__Fusobacteria;c__Fusobacteriia;o__Fusobacteriales;f__Fusobacteriaceae;g__Cetobacterium
6. k__Bacteria;p__Proteobacteria;c__Gammaproteobacteria;o__Betaproteobacteriales;f__Rhodocyclaceae;g__Methyloversatilis
7. k__Bacteria;p__Proteobacteria;c__Gammaproteobacteria;o__Enterobacteriales;f__Enterobacteriaceae;g__Plesiomonas;s__Ambiguous_taxa.
8. k__Bacteria;p__Proteobacteria;c__Gammaproteobacteria;o__Betaproteobacteriales;f__Methylophilaceae;g__Methylophilus
9. k__Bacteria;p__Proteobacteria;c__Alphaproteobacteria;o__Rhizobiales;f__Devosiaceae;g__Devosia.
10. k__Bacteria;p__Bacteroidetes;c__Bacteroidia;o__Chitinophagales;f__Chitinophagaceae;g__Terrimonas.

### Top 5 bacteria families and abundance (% of total counts) in *Proteobacteria* phylum (Fig 4C)

1. k__Bacteria;p__Proteobacteria;c__Gammaproteobacteria;o__Betaproteobacteriales;f__Burkholderiaceae;g__Sphaerotilus.
2. k__Bacteria;p__Proteobacteria;c__Gammaproteobacteria;o__Aeromonadales;f__Aeromonadaceae;g__Aeromonas.
3. k__Bacteria;p__Proteobacteria;c__Gammaproteobacteria;o__Betaproteobacteriales;f__Rhodocyclaceae;g__Methyloversatilis.
4. k__Bacteria;p__Proteobacteria;c__Gammaproteobacteria;o__Enterobacteriales;f__Enterobacteriaceae;g__Plesiomonas;s__Ambiguous_taxa.
5. k__Bacteria;p__Proteobacteria;c__Gammaproteobacteria;o__Betaproteobacteriales;f__Methylophilaceae;g__Methylophilus.

### Top 4 bacteria families and abundance (% of total counts) in *Bacteroidetes* phylum (Fig 4D)

1. k__Bacteria;p__Bacteroidetes;c__Bacteroidia;o__Sphingobacteriales;f__env.OPS_17;g__Ambiguous_taxa;s__Ambiguous_taxa.
2. k__Bacteria;p__Bacteroidetes;c__Bacteroidia;o__Chitinophagales;f__Chitinophagaceae;g__Terrimonas.
3. k__Bacteria;p__Bacteroidetes;c__Bacteroidia;o__Cytophagales;f__Spirosomaceae;g__Emticicia.
4. k__Bacteria;p__Bacteroidetes;c__Bacteroidia;o__Chitinophagales;f__Chitinophagaceae;g__Sediminibacterium

## Supporting information

S1 Data (DOCX)

S1 Checklist (PDF)
