# Correction: Opportunistic gill infection is associated with TiO2 nanoparticle-induced mortality in zebrafish

**DOI:** 10.1371/journal.pone.0301783

**Published:** 2024-04-01

**Authors:** The PLOS One Staff

Fig 4 is incorrect. The authors have provided a corrected version here.

The publisher apologizes for the error.

**Fig 4:**
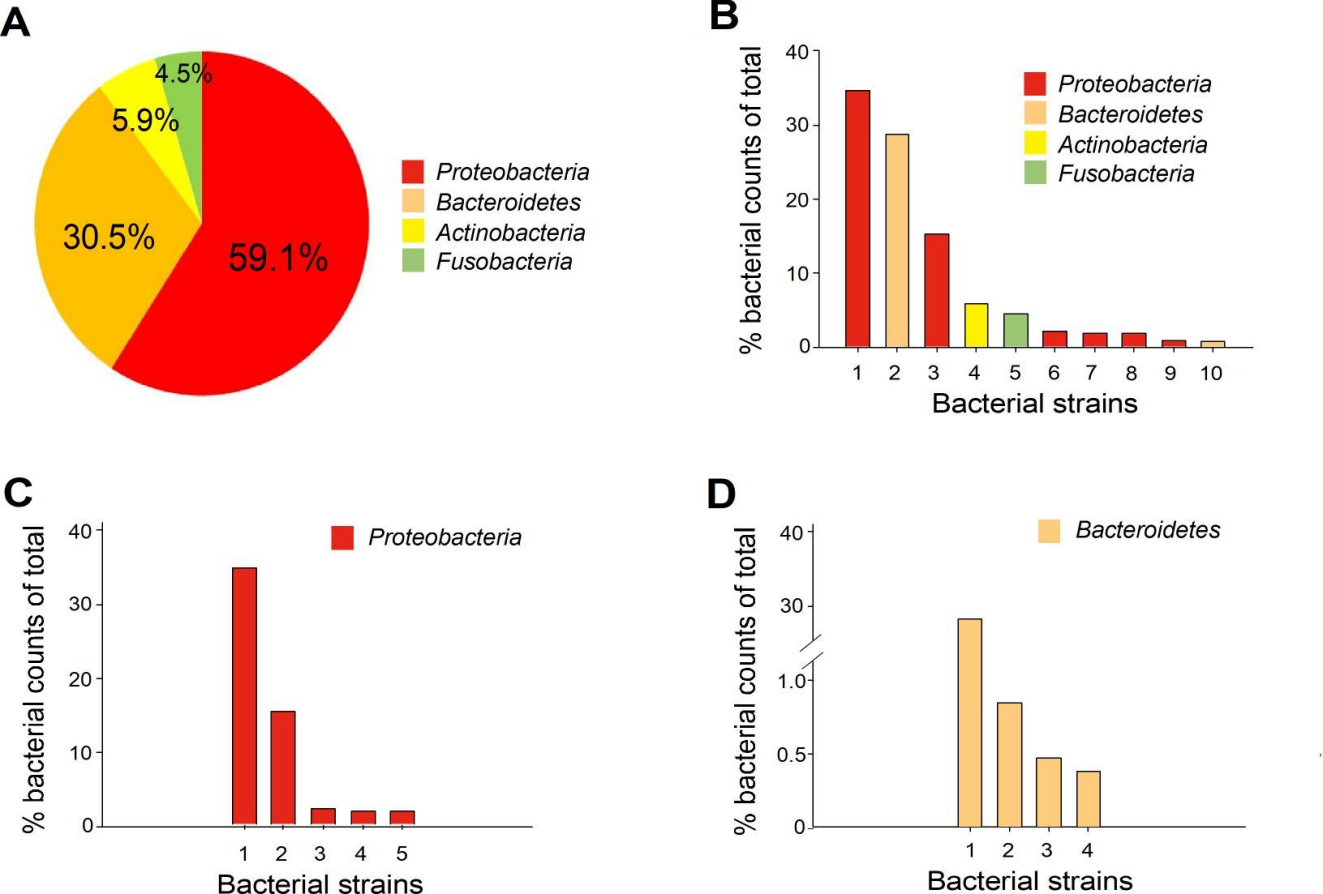
Metagenomic analysis of bacterial communities in the gill samples of zebrafish with TiO2NP-induced injury. (A) Relative abundance (% relative to the total) of the bacteria populations calculated for specific hypervariable regions of 16S ribosomal RNA through new-generation sequencing analyses. (B) Relative abundance (% counts of total) of the top 10 overall bacteria families (listed in the following paragraph). (C) Top 5 bacteria families in the Proteobacteria phylum (most abundant phylum; listed below). (D) Top 4 bacteria families in Bacteroidetes phylum (second abundant phylum; listed below).
