# Supplementary material for: Opportunistic gill infection is associated with TiO2 nanoparticle-induced mortality in zebrafish
Source: PLoS One. 2021 Jul 20;16(7):e0247859. doi: 10.1371/journal.pone.0247859 (PMC8291654; doi:10.1371/journal.pone.0247859)
Supplement: S1 Data — (DOCX) [file pone.0247859.s001.docx]

**S1 Data for:**

**Opportunistic gill infection is associated with TiO_2_ nanoparticle-induced mortality in zebrafish**

Chiao-Yi Huang ^1^, Wei-Sheng Yu ^1,2^, Geng-Chia Liu ^1,2^, Shih-Che Hung ^3^, Jen-Hsiang Chang ^4^, Jen-Che Chang ^1,5^, Chia-Liang Cheng ^6^, Der-Shan Sun ^1^, Ming-Der Lin ^1^, Wen-Ying Lin ^1^, Yin-Jeh Tzeng ^1^ * and Hsin-Hou Chang ^1^ *

^1^ Department of Molecular Biology and Human Genetics, Tzu-Chi University, Hualien, 970, Taiwan.

^2^ Tzu-Chi Senior High School Affiliated with Tzu-Chi University, Tzu-Chi University, Hualien, Taiwan.

^3^ Institute of Medical Sciences, Tzu-Chi University, Hualien, 970, Taiwan.

^4^ Department and Graduate School of Computer Science, National Pingtung University, Pingtung, Taiwan.

^5^ Stella Maris High School, Hualien, Taiwan.

^6^ Department of Physics, National Dong Hwa University, Hualien, 974, Taiwan.

**Running title:** TiO_2_ NPs-induced opportunistic infection in zebrafish

**Key words:** zebrafish, gill, titanium dioxide (TiO_2_), nanoparticle, opportunistic infection, microbiome, microflora, metagenomic analysis, *Proteobacteria*, *Bacteroidetes*, and *Actinobacteria*

* To whom correspondence and reprint requests should be addressed.

Hsin-Hou Chang, Ph.D.

Room D407, Tzu-Chi University, No. 701, Section 3, Chung-Yang Road, Hualien 97004, Taiwan.

Tel: 886-38565301 ext 2667 Fax: 886-38578386

E-mail: [hhchang@mail.tcu.edu.tw](mailto:hhchang@mail.tcu.edu.tw)

Yin-Jeh Tzeng, Ph.D.

Room D431, Tzu-Chi University, No. 701, Section 3, Chung-Yang Road, Hualien 97004, Taiwan.

Tel: 886-38565301 ext 2671 Fax: 886-38578386

E-mail: [tzeng@mail.tcu.edu.tw](mailto:tzeng@mail.tcu.edu.tw)

Page 1: Figure S1

Page 2: Figure S2

**S1 Fig**


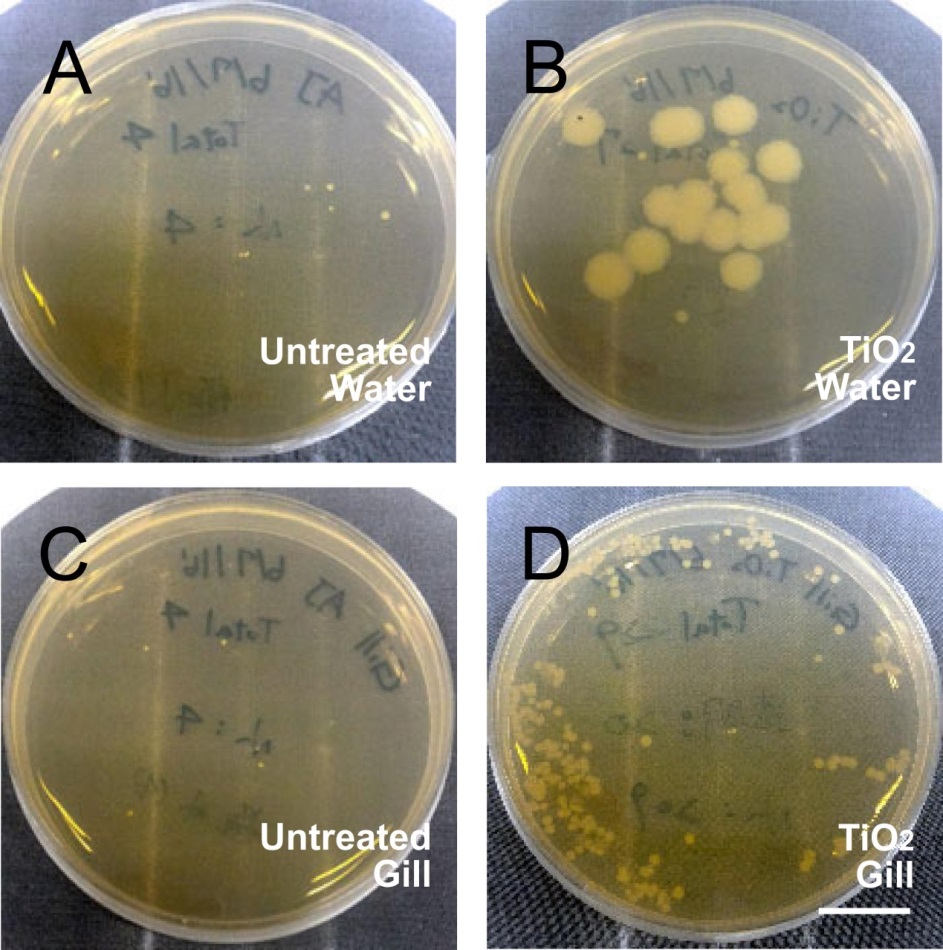


**S1 Fig.** **Example of bacteria colonies obtained from water and zebrafish gill samples.** Representative agar plate images (A-D) revealed bacteria colonies from water (A, B) and fish gill (C, D) samples without (A, C) or with TiO_2_NP (B, D) treatments.

**S2 Fig**


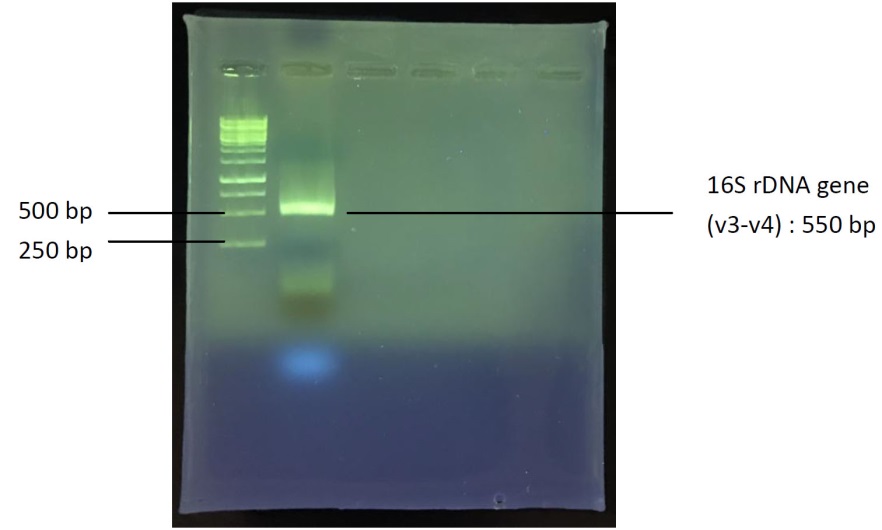


**S2 Fig.** **Image of DNA agarose gel.** A quality control check before metagenomic analysis is showed. Lane 1 displays the DNA size marker, and lane 2 displays the 16S rRNA DNA fragment (v3-v4) 550 base pairs, are indicated, respectively.
